# Maternal occupational exposure to solvents and gastroschisis in offspring - National Birth Defects Prevention Study 1997–2011

**DOI:** 10.1136/oemed-2019-106147

**Published:** 2020-01-16

**Authors:** Nynke Spinder, Lynn M Almli, Tania A Desrosiers, Kathryn E Arnold, Jorieke E H Bergman, Hans Kromhout, H Marike Boezen, Hermien E K de Walle, Carissa Rocheleau, Jennita Reefhuis

**Affiliations:** 1 National Center on Birth Defects and Developmental Disabilities, Centers for Disease Control and Prevention, Atlanta, Georgia, United States; 2 Department of Epidemiology, University of Groningen, University Medical Center Groningen, Groningen, Netherlands; 3 Department of Genetics, Univeristy of Groningen, University Medical Center Groningen, Groningen, Netherlands; 4 Gillings School of Global Public Health, Department of Epidemiology, University of North Carolina, Chapel Hill, North Carolina, United States; 5 Institute for Risk Assessment Sciences, Division of Environmental Epidemiology, Utrecht University, Utrecht, Netherlands; 6 Groningen Research Institute for Asthma and COPD (GRIAC), University of Groningen, University Medical Center Groningen, Groningen, Netherlands; 7 National Institute for Occupational Safety and Health, Centers for Disease Control and Prevention, Cincinnati, Ohio, United States

**Keywords:** gastroschisis, maternal, occupational, periconceptional, Solvents

## Abstract

**Objectives:**

The aim of this study was to assess the association between maternal occupational exposure to solvents and gastroschisis in offspring.

**Methods:**

We used data from the National Birth Defects Prevention Study, a large population-based case-control study of major birth defects conducted in 10 US states from 1997 to 2011. Infants with gastroschisis were ascertained by active birth defects surveillance systems. Control infants without major birth defects were selected from vital records or birth hospital records. Self-reported maternal occupational histories were collected by telephone interview. Industrial hygienists reviewed this information to estimate exposure to aromatic, chlorinated and petroleum-based solvents from 1 month before conception through the first trimester of pregnancy. Cumulative exposure to solvents was estimated for the same period accounting for estimated exposure intensity and frequency, job duration and hours worked per week. ORs and 95% CIs were estimated to assess the association between exposure to any solvents or solvent classes, and gastroschisis risk.

**Results:**

Among 879 cases and 7817 controls, the overall prevalence of periconceptional solvent exposure was 7.3% and 7.4%, respectively. Exposure to any solvent versus no exposure to solvents was not associated with gastroschisis after adjusting for maternal age (OR 1.00, 95% CI 0.75 to 1.32), nor was an association noted for solvent classes. There was no exposure-response relationship between estimated cumulative solvent exposure and gastroschisis after adjusting for maternal age.

**Conclusion:**

Our study found no association between maternal occupational solvent exposure and gastroschisis in offspring. Further research is needed to understand risk factors for gastroschisis.

**Key messages**

**What is already known about this subject?**

- Gastroschisis is a severe abdominal wall defect with increasing prevalence and largely unknown aetiology. One previous study suggested that occupational exposure to solvents might be associated with gastroschisis.

**What are the new findings?**

- We evaluated the association between maternal occupational exposure to solvents and gastroschisis in offspring in a large population-based case-control study of major birth defects, and did not find an association between solvent exposure and gastroschisis.

**How might this impact on policy or clinical practice in the foreseeable future?**

- These results do not explain the increase in gastroschisis that has been observed over the past few decades, and has therefore no impact on policy or clinical practice. Continued exploration of risk factors or a combination of risk factors for gastroschisis is warranted.

## Introduction

Gastroschisis is a severe birth defect of the abdominal wall, which involves a full-thickness paraumbilical defect through which intestines and other organs may herniate without a covering membrane. Gastroschisis is most often an isolated defect and is not associated with chromosomal disorders.^1^ The prevalence of gastroschisis in the USA is increasing, and is currently estimated to be approximately 4.5 per 10 000 births.^2^ The majority of infants need surgery to close the abdominal wall. After surgery, 90% of these infants are alive at 1 year of age.^3^

The aetiology of gastroschisis is unknown and much debated. One recent hypothesis is that gastroschisis develops due to rupture or non-closure of the membrane covering the umbilical ring between 8 and 11 weeks after fertilisation^4 5^; however, other hypotheses are suggested.^6 7^ The increased prevalence of gastroschisis suggests a role of unknown environmental factors, which might have an effect on the developing membrane of the umbilical ring.^8^ Epidemiological studies show that the strongest risk factor for gastroschisis is young maternal age (<20 years of age).^9^ Other risk factors associated with gastroschisis are maternal smoking,^9^ alcohol consumption, illicit drugs^10 11^ and low maternal body mass index (BMI).^12^ Maternal illnesses such as depression, urinary tract infections and sexually transmitted diseases before or early in pregnancy^8 13–15^; and use of specific medications early in pregnancy^9 13 16^ have also been associated with gastroschisis. The relationships between these risk factors and gastroschisis are complicated by young maternal age, since it is not clear whether maternal age is a confounder or on causal pathways involving these exposures and gastroschisis.

Fewer studies have examined the role of occupational exposures that might be associated with gastroschisis. Millions of workers in the USA are exposed to solvents, which are present in paints, adhesives, glues and degreasing/cleaning agents. Solvents are used for production of plastics, textiles, printing inks, agricultural products and pharmaceuticals.^17^ Solvents are known for their reproductive toxicity,^18^ and might therefore have an effect on the development of gastroschisis. A recent meta-analysis found that maternal occupational exposure to solvents before and during pregnancy is associated with several birth defects, including neural tube defects, congenital heart defects and oral facial clefts.^19^ One case-control study was conducted assessing maternal occupational exposure to solvents and gastroschisis. This study, including 110 gastroschisis cases and 220 controls, reported an association (OR 2.55, 95% CI 1.10 to 5.89).^20^

The aim of our study was to assess the association between estimated maternal occupational exposure to solvents during the periconceptional period (1 month before conception through 3 months after conception) and gastroschisis in offspring using data from the National Birth Defects Prevention Study (NBDPS).

## Methods

### Study design

The NBDPS is a large population-based, multicenter, case–control study of major structural birth defects in the USA. Detailed information about NBDPS has been previously described.^21^ In brief, pregnancies with estimated delivery dates between 1 October, 1997, and 31 December, 2011, in Arkansas, California, Georgia, Iowa, Massachusetts, North Carolina, New Jersey, New York, Texas and Utah were included.

All states included liveborn cases, whereas most states also included cases among stillbirths (death after >20 of gestational age) and terminated pregnancies with a prenatal diagnosis of birth defects. Cases were ascertained by the participating states’ birth defects surveillance systems up to 2 years after delivery. To confirm eligibility, clinical information abstracted from medical records was reviewed by a clinical geneticist at each center using a systematic study-wide classification protocol. Only one infant per family was eligible for the study. Controls were liveborn infants without major birth defects selected randomly from either vital records or birth hospital records from the same geographical regions and time-period as cases. All participants gave informed consent.

### Case classification

Cases were classified as ‘isolated’ if they had one major defect or two major defects involving the same organ system; cases were classified as ‘multiple’ if they had multiple major defects in different organ systems.^22^ Infants were excluded if defects were related to a single gene condition or a chromosomal abnormality, or if case information was classified as limb-body wall complex or amniotic band sequence. Furthermore, infants with a first-degree family member with gastroschisis were excluded because of unknown heredity.

### Exposure assessment

Women who participated in the NBDPS completed a computer assisted telephone interview in English or Spanish between 6 weeks and 24 months after the estimated delivery date. Mothers were asked to report information about demographics, medication use and lifestyle during pregnancy and the 3 months preceding pregnancy. Occupational histories were collected among women who reported a job for at least 1 month or more during the 3 months prior to conception through the end of pregnancy. Women were asked about their job title, employer name, what the company makes or does, their primary tasks and duties, description of chemicals and machines handled on the job, dates of employment and hours and days worked per week for each job.

All jobs were coded using the Standard Occupational Classification (SOC) 2010.^23^ Industrial hygienists and occupational experts working at the National Institute for Occupational Safety and Health performed, blinded by case–control status, a retrospective exposure assessment for a variety of occupational exposures, including 10 solvents: benzene, xylene, toluene, carbon tetrachloride, chloroform, methylene chloride, perchloroethylene, 1,1,1-trichloroethane, trichloroethylene and Stoddard solvent. Each job was assigned scores for estimated relative intensity of exposure (online supplementary table 1) and frequency (none, >0 to <2 hours per week, 2 to 10 hours per week, 11 to 19 hours per week, >19 hours per week exposed in a standard 40-hour week), as well as probability and confidence scores to reflect the certainty of the raters. Probability score was defined as the estimated percentage of mothers with similar jobs being exposed to solvents (<10% to >90%). Confidence score was defined as the confidence of the industrial hygienist that mothers’ job matched the job description indicating solvent exposure (low to very high). Raters compiled previously published exposure measurements from a variety of studies and workplace evaluations to guide them as they assigned ratings. If ratings between the hygienists disagreed, they met with an additional industrial hygienist/occupational health expert to discuss and reach consensus on the most appropriate rating.

10.1136/oemed-2019-106147.supp1 Supplementary data

To combine information on intensity and frequency of exposure, as well as self-reported hours worked per week and duration of the job during the window of biological interest, intensity and frequency scores were quantitatively mapped to the midpoint of their estimated range and calculated as follows: (intensity)×(frequency as hours per week/40 hours per week)×((self-reported work frequency (hours/week))/(7 days/week))×(number of days worked during the periconceptional period). This resulted in an estimated cumulative exposure (in parts per million (ppm)-hours or µg/m^3^) for each job during the periconceptional period^24^; a similar approach has been described and used elsewhere.^25^

Although most mothers held one job, some mothers held multiple jobs during the periconceptional period. Mothers with multiple jobs were considered as exposed if any of her jobs during the periconceptional period was rated as exposed. If all jobs were rated as unexposed, mothers were considered to have been unexposed. The estimated cumulative exposure (ppm-hours or µg/m^3^) was summed across all jobs. Mothers who reported not being employed during the periconceptional period were excluded from this analysis to reduce the potential for bias due to work status or employment-related factors.^26^

### Statistical analysis

Frequency distributions of maternal demographic and behavioural characteristics were calculated for cases and controls. Additionally, frequency distributions for solvent-exposed and solvent-unexposed controls were calculated to give an overview of characteristics for the working population. The prevalence of 23 SOC major job groups for solvent-exposed and solvent-unexposed case and control mothers was tabulated to characterise the occupation types held in our exposed study population.

Correlations between exposure status within and between solvent classes were explored in mothers of controls to determine the best modelling strategy. Solvents were evaluated individually and subsequently grouped by class into aromatic solvents (benzene, xylene, toluene) and chlorinated solvents (carbon tetrachloride, chloroform, methylene chloride, perchloroethylene, 1,1,1-trichloroethane, trichloroethylene) due to high correlation within these groupings. For example, 98% of mothers exposed to trichloroethylene were also considered to be exposed to methylene chloride (n=259). Correlation between assigned solvent classes was substantially lower compared with correlation between individual chemicals within solvent classes (online supplementary table 2).

The prevalence of occupational exposure (no exposure/exposure) was estimated for any solvent exposure and solvent classes (aromatic, chlorinated and Stoddard solvents). Univariate logistic regression analyses were performed to estimate ORs and 95% CIs in order to assess the association between maternal occupational exposure to solvents and gastroschisis, using non-exposed mothers for the solvent class under analysis as the reference category. Sparse data (≤3 exposed individuals) were not presented, and ORs were not estimated. To assess covariates associated with gastroschisis and/or solvent exposure for the multivariate regression analyses, we introduced one covariate at a time into the model. At least a 10%-point difference in the OR for the main effect between solvents and gastroschisis was considered as a meaningful difference. We examined the following self-reported covariates: NBDPS center, maternal education (≤12 and >12 years), race/ethnicity (non-Hispanic white, non-Hispanic black, Hispanic and other), BMI (continuous), parity (0 and ≥1), maternal cigarette smoking including secondhand smoke at work or at home (yes/no), alcohol intake (yes/no), illicit drug use (yes/no) during the periconceptional period. None of these covariates produced a 10%-point difference in the OR for the main effect. Maternal age was *a priori* selected as covariate, due to the strong association between young maternal age and gastroschisis.

Sensitivity analyses were conducted in order to account for exposure misclassifications. First we repeated analyses restricting the exposed group to women with at least one job with an estimated probability of exposure ≥10%. Second, we repeated the solvent-gastroschisis analyses restricting to women with at least one job with medium/high confidence. Mothers with multiple jobs that changed exposure category due to those restrictions were excluded from analyses.

Because young maternal age is the strongest risk factor for gastroschisis, analyses stratified by maternal age (<20 and ≥20 years) were conducted. Furthermore, stratified analyses were conducted for isolated and multiple defects, since isolated and non-isolated defects may differ in aetiology.

Exposure-response analyses for overall solvent exposure and each solvent class were conducted to assess cumulative maternal occupational solvent exposure and gastroschisis. The estimated cumulative exposure was analysed in four groups, based on tertiles of the exposed controls (none, level 1, 2 and 3). Crude and adjusted ORs (aORs) and 95% CIs were estimated for the association between cumulative exposure to any solvents and classes and gastroschisis. Logistic regression was used to test for a linear trend in the betas of the tertiles of cumulative solvent exposure using the Wald test of significance. Separate analyses were conducted for intensity and frequency of exposure.

## Results

In total, 13 279 control infants or infants with gastroschisis were identified. One infant with an amniotic band sequence/limb-body wall complex was excluded. There were 4573 mothers excluded because no job was reported during the periconceptional period. They were homemakers (n=2838), students (n=617), disabled (n=45), in between jobs (n=182), not specified (n=35) or were missing information about employment (n=485). Finally, 369 mothers were excluded because their reported job was not held during the periconceptional period or because exposure could not by assigned (n=2). Four cases and five controls were excluded because they had a first-degree relative with gastroschisis. In total, 879 infants with gastroschisis and 7817 control infants were included in this study.

The comparisons of maternal characteristic between cases and controls are shown in table 1. Mothers of cases with gastroschisis were younger, had fewer years of education, were more likely to be Hispanic and had a lower BMI. Mothers of cases had greater exposure to cigarette smoking, and used alcohol and illicit drugs more frequently during the periconceptional period compared with mothers of controls. Exposed mothers had significantly fewer years of education, had greater exposure to cigarette smoking, but consumed less alcohol than non-exposed mothers (online supplementary table 3). Among cases, 96.2% were live births, 3.1% were fetal deaths (>20 weeks of gestational age) and 0.7% were induced abortions.

**Table 1:** Baseline characteristics of gastroschisis cases and controls, National Birth Defects Prevention Study, USA, 1997–2011

	Gastroschisis cases (n=879)	Total controls (n=7817)
	N (%)	N (%)
Maternal age at delivery (years)*		
<20	246 (28.0%)	492 (6.3%)
20–24	408 (46.4%)	1747 (22.3%)
25–29	161 (18.3%)	2240 (28.7%)
30–34	52 (5.9%)	2163 (27.7%)
≥35	12 (1.4%)	1176 (15.0%)
Maternal education*		
≤12 years	498 (56.9%)	2504 (32.1%)
>12 years	377 (43.1%)	5300 (67.9%)
Maternal race-ethnicity*		
Non-Hispanic white	503 (57.2%)	5003 (64.0%)
Non-Hispanic black	83 (9.4%)	899 (11.5%)
Hispanic	223 (25.4%)	1433 (18.3%)
Other	70 (8.0%)	482 (6.2%)
Pre-pregnancy BMI (kg/m^2^)*		
Underweight (<18.5)	63 (7.3%)	352 (4.6%)
Normal weight (18.5–25)	603 (69.6%)	4125 (53.9%)
Overweight (25-30)	159 (18.3%)	1772 (23.2%)
Obese (>30)	42 (4.8%)	1403 (18.3%)
Parity*		
0	611 (69.5%)	3514 (45.0%)
≥1	268 (30.5%)	4301 (55.0%)
Maternal cigarette smoking during periconceptional period*†		
Yes	485 (55.2%)	2498 (32.0%)
No	391 (44.8%)	5302 (68.0%)
Maternal alcohol use during periconceptional period*		
Yes	429 (48.9%)	3327 (42.7%)
No	448 (51.2%)	4464 (56.7%)
Maternal illicit drug use during periconceptional period*‡		
Yes	117 (13.3%)	329 (4.2%)
No	761 (86.7%)	7486 (95.8%)

Totals do not add up due to missing data.

*Significant difference between cases and controls (p value <0.05) using X^2^ tests.

†Self-reported cigarette smoking and secondhand cigarette smoke exposure at work and at home.

‡Included marijuana, hash, cocaine, crack, hallucinogens, heroin and mushrooms.

BMI, body mass index;

The prevalence of estimated occupational exposure to any solvent during the periconceptional period was 7.3% among cases and 7.4% among controls (table 2). Mothers with exposure to any solvents worked in production occupations (28.0%), personal care and service occupations (18.4%), building and grounds cleaning and maintenance occupations (12.9%). There was no association between maternal occupational exposure to solvents and gastroschisis (aOR 1.00, 95% CI 0.75 to 1.32, adjusted for maternal age) (table 2). Exposure prevalence for aromatic solvents was 2.2% for cases and 2.1% for controls, and there was no association between aromatic solvents and gastroschisis (aOR 1.15, 95% CI 0.69 to 1.92). Exposure to chlorinated solvents was most common; 6.4% for both cases and controls. However, no increased OR was identified in association with gastroschisis (aOR 0.98, 95% CI 0.73 to 1.32). The prevalence of Stoddard solvents exposure was 2.2% for cases and 2.0% for controls, but no association between Stoddard solvents exposure and gastroschisis was found (aOR 0.84, 95% CI 0.51 to 1.39). When analyses were restricted to jobs with an estimated exposure probability ≥10%, similar results were observed compared with analyses that included all women (data not shown). In addition, analyses restricted to jobs with medium and high confidence of solvent exposure also showed similar results (data not shown).

**Table 2:** Prevalence of estimated maternal occupational exposure to solvents during the periconceptional period* and risk of gastroschisis in offspring, National Birth Defects Prevention Study, USA, 1997–2011

Solvent classes	Gastroschisis cases (n=879)	Total controls (n=7817)	Unadjusted		Adjusted†	
	N (%)	N (%)	OR	95% CI	OR	95% CI
Any solvent						
No exposure	813 (92.7%)	7233 (92.6%)	Ref		Ref	
Exposure	64 (7.3%)	579 (7.4%)	0.98	0.75 to 1.29	1.00	0.75 to 1.32
Aromatic solvents						
No exposure‡	859 (97.8%)	7651 (97.9%)	Ref		Ref	
Exposure	19 (2.2%)	163 (2.1%)	1.04	0.64 to 1.68	1.15	0.69 to 1.92
Chlorinated solvents						
No exposure‡	821 (93.6%)	7311 (93.6%)				
Exposure	56 (6.4%)	502 (6.4%)	0.98	0.75 to 1.32	0.98	0.73 to 1.32
Stoddard solvents						
No exposure‡	858 (97.8%)	7658 (98.0%)	Ref		Ref	
Exposure	19 (2.2%)	158 (2.0%)	1.07	0.66 to 1.74	0.84	0.51 to 1.39

Totals do not add up due to missing data.

*One month before conception through 3 months after conception.

†Adjusted for maternal age at delivery as a continuous variable (no missing values).

‡No exposure for outcome under analysis.

Analysis stratified by maternal age at delivery (<20 and ≥20 years of age) showed that exposure to solvents was more prevalent among cases with older mothers (8.7%) compared with cases with younger mothers (3.7%) (data not shown). The OR for any solvent exposure versus no solvent exposure for older mothers showed no significant increase (OR 1.16, 95% CI 0.87 to 1.55), nor were increased ORs observed for solvent classes. The OR for any solvents among younger mothers showed no increase (OR 0.74, 95% CI 0.34 to 1.61). No increased ORs were found for solvents by class.

Stratified analysis by isolated and multiple defects included 801 cases with an isolated defect and 78 cases with multiple defects (table 3). Exposure to any solvent was more common among exposed cases with multiple defects (14.1%) compared with exposed cases with isolated defects (6.6%). An increased OR was found for any solvent exposure (aOR 2.11, 95% CI 1.10 to 4.06) for infants with multiple defects. The estimate was lower for chlorinated solvents (aOR 1.44, 95% CI 0.65 to 3.17). The ORs for aromatic and Stoddard solvents could not be calculated due to sparse data (n≤3). Increased ORs were not observed for isolated defects (eg, any solvent exposure vs no solvent: aOR 0.90, 95% CI 0.66 to 1.22).

**Table 3:** Prevalence of estimated maternal occupational exposure to solvents during the periconceptional period^a^ and risk of gastroschisis in offspring, stratified by isolated vs multiple defects, National Birth Defects Prevention Study, USA, 1997–2011

Solvent classes	Isolated defects (cases n=801/controls n=7817)						Multiple defects (cases n=78/controls n=7817)					
	Exposed		Crude		Adjusted†		Exposed		Crude		Adjusted†	
	Cases	Controls	OR	95% CI	aOR	95% CI	Cases	Controls	OR	95% CI	aOR	95% CI
Any solvent	53 (6.6%)	579 (7.4%)	0.89	0.66 to 1.19	0.90	0.66 to 1.22	11 (14.1%)	579 (7.4%)	2.05	1.08 to 3.90	2.11	1.10 to 4.06
Aromatic solvents	16 (2.0%)	163 (2.1%)	0.96	0.57 to 1.61	1.05	0.61 to 1.81	<3		NC		NC	
Chlorinated solvent	49 (6.1%)	502 (6.4%)	0.95	0.70 to 1.29	0.94	0.69 to 1.29	7 (9.0%)	502 (6.4%)	1.44	0.66 to 3.14	1.44	0.65 to 3.17
Stoddard solvents	18 (2.3%)	158 (2.0%)	1.12	0.68 to 1.83	0.87	0.52 to 1.46	≤3		NC		NC	

*One month before conception through 3 months after conception.

†Adjusted for maternal age at delivery as a continuous variable (no missing values).

NC, not calculated due to sparse data (n≤3 individuals).

The prevalence and ORs for the estimated maternal cumulative exposure to solvents during the periconceptional period and gastroschisis in offspring are shown in table 4. We did not observe an exposure level-response association for any solvent exposure, nor for aromatic, chlorinated or Stoddard solvents exposure. No trends were observed for increasing cumulative maternal occupational exposures to solvents or to solvent classes. Exposure-response analyses could not be performed for multiple defects, due to too few cases per category. Separate analyses for intensity and frequency of exposure showed no differences between lower and higher intensities or frequencies of exposure (online supplementary tables 4 and 5).

**Table 4:** Prevalence of cumulative maternal occupational exposure to solvents during the periconceptional period* and risk of gastroschisis in offspring, National Birth Defects Prevention Study, USA, 1997–2011

Solvent classes§	Cases (n=879)†	Controls (n=7817)†	Unadjusted		Adjusted‡	
	N %	N %	OR	95% CI	OR	95% CI
Any solvents			*P_trend_*=0.68		*P_trend_*=0.79	
No exposure¶	813 (92.7%)	7233 (92.6%)	Ref		Ref	
Level 1	14 (1.6%)	193 (2.5%)	0.64	0.37 to 1.11	0.68	0.38 to 1.20
Level 2	27 (3.1%)	191 (2.4%)	1.26	0.83 to 1.89	1.37	0.89 to 2.12
Level 3	23 (2.6%)	194 (2.5%)	1.05	0.68 to 1.63	0.96	0.60 to 1.51
Aromatic solvents			*P_trend_*=0.69		*P_trend_*=0.66	
No exposure¶	859 (97.8%)	7651 (97.9%)	Ref		Ref	
Level 1	5 (0.6%)	54 (0.7%)	0.83	0.33 to 2.07	1.08	0.41 to 2.84
Level 2	7 (0.8%)	54 (0.7%)	1.16	0.52 to 2.55	1.50	0.64 to 3.52
Level 3	7 (0.8%)	54 (0.7%)	1.16	0.52 to 2.55	0.98	0.43 to 2.24
Chlorinated solvents			*P_trend_*=0.58		*P_trend_*=082	
No exposure¶	821 (93.6%)	7311 (93.6%)	Ref		Ref	
Level 1	11 (1.3%)	167 (2.1%)	0.59	0.32 to 1.08	0.61	0.32 to 1.15
Level 2	24 (2.7%)	167 (2.1%)	1.28	0.83 to 1.98	1.41	0.89 to 2.24
Level 3	21 (2.4%)	167 (2.1%)	1.12	0.71 to 1.77	0.97	0.59 to 1.54
Stoddard solvents			*P_trend_*=0.95		*P_trend_*=0.37	
No exposure¶	858 (97.8%)	7658 (98.0%)	Ref		Ref	
Level 1	7 (0.8%)	51 (0.7%)	1.23	0.55 to 2.71	0.93	0.41 to 2.14
Level 2	8 (0.9%)	54 (0.7%)	1.32	0.63 to 2.79	1.09	0.50 to 2.38
Level 3	4 (0.5%)	53 (0.7%)	0.67	0.24 to 1.87	0.52	0.18 to 1.48

*P_trend_* = Wald p value for testing linear trend of the tertile betas.

*One month before conception through 3 months after conception.

†Missing cases/controls varied from four to seven mothers across exposures because exposure could not be assigned or cumulative exposure could not be calculated.

‡Adjusted for maternal age at delivery as a continuous variable (no missing values).

§Based on tertiles of the exposed controls.

¶No exposure for outcome under analysis.

## Discussion

In this study we did not find an association between maternal occupational exposure to chlorinated, aromatic or Stoddard solvents during the periconceptional period and isolated gastroschisis in offspring. We did observe an association between exposure to any solvents and gastroschisis co-occurring with other defects, but this should be interpreted with caution. The observed association did not reach statistical significance for aromatic and chlorinated solvents, but these analyses were based on a small number of multiple cases. Overall, the power of these analyses is limited; only 78 cases were included, of which 11 cases were exposed. Furthermore, gastroschisis is mainly known as an isolated defect. When we further explored the types of multiple defects in our study population, we did not identify a specific pattern among the defects in association with the gastroschisis. Most cases had one additional birth defect, such as a congenital heart defect or a neural tube defect, which have been previously associated with occupational solvent exposure.^19^

Stratification by maternal age showed no association between occupational exposure to solvents and gastroschisis. No exposure-response relationship for any solvents or solvent classes and gastroschisis were found.

One previous study reported an association between maternal occupational exposure to solvents and gastroschisis (OR 2.55, 95% CI 1.10 to 5.89).^20^ This case–control study was performed by the California Birth Defects Monitoring Program in 1989 and 1990. Case/control ascertainment and inclusion criteria were comparable to the NBDPS. In this study by Torfs and colleagues, during an interview mothers were asked to describe any occupations performed, including specific tasks, during the 3 months before conception and the first trimester. One industrial hygienist, blinded by outcome, evaluated the type of exposure that was associated with the job. Solvent types included aromatic hydrocarbons, gaseous aliphatic hydrocarbons and liquid aliphatic hydrocarbons. Exposure assessment was comparable to our exposure assessment. However, we used a multiple expert rater method of exposure assessment, which is known to reduce exposure misclassification.^27^ The prevalence of exposure was not reported for occupational exposure specifically, and could therefore not be compared with our exposure prevalence. Finally, we included 879 cases with gastroschisis whereas Torfs and colleagues included only 150 cases. Their study did not report on whether cases had isolated defects or multiple defects including gastroschisis. Therefore, our results regarding multiple defects could not be compared. In conclusion, differences in results could be explained by different inclusion criteria, possible exposure misclassification and a difference in power.

### Strengths and limitations

One strength of this study is that we used data from the NBDPS, a large population-based case–control study in which 10 centers participated for most of the study period. Each center covered a birth population between 35 000 and 80 000 births per year.^21^ Therefore, a relatively large number of infants with gastroschisis could be included. Live births, stillbirths and terminated pregnancies were included in most states, thereby mitigating selection bias due to survival. In addition, careful clinical review and classification by clinical geneticists were conducted, reducing outcome misclassification. Finally, the NBDPS included control infants without major birth defects. These infants were generally representative of the base population from which they were selected.^28^

Another strength of this study is that we restricted our study sample to women who reported having a job during the periconceptional period. This is important because employment status is related to sociodemographic and (reproductive) health characteristics that are generally recognised risk factors for adverse pregnancy outcomes. By restricting our analyses to employed women, we controlled for confounding by employment status and related factors.^26 29^ The inter-rater reliability of exposure assessment used in this study was fair-to-good and was generally comparable to or slightly higher than reliability estimates from similar studies, therefore it might be less likely that exposure misclassification impacted our results.^24^

Despite our large study sample, the number of exposed cases was relatively low (7%) compared with other population-based studies of occupational solvent exposure during pregnancy (10% to 19%)^19^ using similar exposure assessment methods. This could have resulted in imprecision of our estimates. This is especially true for the exposure-response analyses where less than 3% of exposed cases per level were included. With three levels of exposure, we created a contrast between low and high exposure; however, this resulted in lower power compared with the analysis with two exposure categories. Our estimates were generally more precise than the previous study,^20^ likely due to the unprecedented number of cases available in NBDPS. However, direct comparison to previous work is tenuous given the differences in exposure assessment methodologies. Most women in this population-based study were exposed to relatively low estimated doses of solvents. However, we cannot rule out effects among workers with much higher doses of exposures.

A limitation of exposure assessment is that non-differential misclassification of exposure could have occurred, because assessment was indirect and retrospective. We possibly reduced potential misclassification by looking only at solvent class and not at individual solvents. The sensitivity and specificity of exposure assessment by industrial hygienist is unknown compared with true exposure, since there was no validation by direct exposure measurement. Another limitation of retrospective exposure assessment is the possibility that women avoided or were restricted by their employer to handle certain solvents during work, or wore protective equipment because they wanted to become pregnant or knew they were pregnant.

A limitation of the NBDPS is that selection bias could have occurred, since approximately two-thirds of invited women participated (65% for cases and controls).^21^ However, a previous study showed that NBDPS participants held a wide variety of occupations.^30^

## Conclusion

We did not observe an association between gastroschisis in offspring and estimated maternal occupational exposure to solvents and solvent classes during the periconceptional period in this large population-based case–control study. Among mothers with gastroschisis cases with multiple defects, an association with maternal occupational exposure to solvents was observed, but these results should be interpreted with caution. No exposure-response relationship was observed using estimated cumulative occupational exposure to solvents. Continued exploration of risk factors for gastroschisis is warranted.
