# Supplementary material for: Maternal occupational exposure to solvents and gastroschisis in offspring - National Birth Defects Prevention Study 1997–2011
Source: Occup Environ Med. 2020 Jan 16;77(3):172–8. doi: 10.1136/oemed-2019-106147 (PMC7035687; doi:10.1136/oemed-2019-106147)
Supplement: Supplementary data [file oemed-2019-106147supp001.pdf]

Table 1 Intensity scores for individual solvents

	**Exposure intensity in ppm (Stoddard solvents in mg/m^3)**			
**Solvent classes**	**Very low**	**Low**	**Medium**	**High**
Aromatic solvents				
Benzene	<0.01	0.01 ≤ 0.1	0.1 ≤ 1	≥ 1
Toluene	<0.35	0.35 ≤ 3.5	3.5 ≤ 30	≥ 30
Xylene	<1	1 ≤ 5	5 ≤ 10	≥ 10
Chlorinated solvents				
Carbon tetrachloride	<0.1	0.1 ≤ 1	1 ≤ 10	≥ 10
Chloroform	<0.6	0.6 ≤ 6	6 ≤ 30	≥ 30
Methylene chloride	<0.35	0.35 ≤ 3.5	3.5 ≤ 30	≥ 30
Perchloroethylene	<0.2	0.2 ≤ 2	2 ≤ 15	≥ 15
1,1,1-trichloroethane	<6	6 ≤ 60	60 ≤ 300	≥ 300
Trichloroethylene	<0.35	0.35 ≤ 3.5	3.5 ≤ 30	≥ 30
Stoddard solvents	<6	6 ≤ 60	60 ≤ 300	≥ 300

Table 2 Correlation between solvent classes assessed in controls only

**Solvent classes**	Aromatic solvents	Chlorinated solvents	Stoddard solvents
Aromatic solvents	1	0.38	0.36
Chlorinated solvents	0.38	1	0.43
Stoddard solvents	0.36	0.43	1

Correlation coefficient assessed with Spearman’s rho.

Table 3 Baseline characteristics of exposed and non-exposed control infants, National Birth Defects Prevention Study, USA, 1997-2011

	**Exposed controls (n= 579)**		**Non-exposed controls (n = 7238)**	
	N	(%)	N	(%)
Maternal age at delivery (years) ᶲ				
<20	24	(4.1%)	467	(6.5%)
20-24	152	(26.3%)	1594	(22.0%)
25-29	184	(31.8%)	2053	(28.4%)
30-34	148	(25.6%)	2015	(27.8%)
≥35	71	(12.3%)	1105	(15.3%)
Maternal education ᶲ				
≤12 years	236	(40.8%)	2265	(31.4%)
>12 years	342	(59.2%)	4955	(68.6%)
Maternal race-ethnicity				
Non-Hispanic white	359	(62.0%)	4641	(64.2%)
Non-Hispanic black	68	(11.7%)	831	(11.5%)
Hispanic	106	(18.3%)	1325	(18.3%)
Other	46	(7.9%)	436	(6.0%)
Pre-pregnancy BMI (kg/m^2)				
Underweight (<18.5)	22	(3.9%)	329	(4.6%)
Normal weight (18.5-25)	331	(59.1%)	3792	(53.5%)
Overweight (25-30)	119	(21.3%)	1652	(23.3%)
Obese (>30)	88	(15.7%)	1314	(18.5%)
Parity ᶲ				
0	237	(40.9%)	3277	(45.3%)
≥1	342	(59.1%)	3942	(54.7%)
Maternal cigarette smoking during periconceptional period[a] ᶲ				
Yes	229	(39.6%)	2268	(31.4%)
No	350	(60.4%)	4963	(68.6%)
Maternal alcohol use during periconceptional period ᶲ				
Yes	229	(39.6%)	2268	(31.4%)
No	350	(60.4%)	4948	(68.6%)
Maternal illicit drug use during periconceptional period[b]				
Yes	31	(5.4%)	298	(4.1%)
No	548	(94.6%)	6933	(95.9%)

Totals do not add up due to missing data. BMI = body mass index. [a] = self-reported cigarette smoking and second-hand cigarette smoke exposure at work and at home, [b] = included marijuana, hash, cocaine, crack, hallucinogens, heroin, and mushrooms, ᶲ = significant difference between exposed and non-exposed controls (p-value <0.05) using Chi Square tests.

Table 4 Estimated intensity of maternal occupational solvents exposure during the periconceptional period[a] and risk of gastroschisis in offspring, National Birth Defects Prevention Study, USA, 1997-2011

	**Gastroschisis cases (n= 879)**		**Total Controls (n = 7817)**		**Unadjusted**		**Adjusted[b]**	
**Solvent classes**	N	(%)	N	(%)	OR	95% CI	OR	95% CI
Any solvent								
No exposure	823	(93.6%)	7359	(94.1%)	Ref		Ref	
Very low	28	(3.2%)	225	(2.9%)	1.11	0.75 – 1.66	1.22	0.80 – 1.86
Low	19	(2.2%)	118	(1.5%)	1.44	0.88 – 2.35	1.17	0.70 – 1.97
Medium	7	(0.8%)	103	(1.3%)	0.61	0.28 – 1.31	0.69	0.31 – 1.53
High	<3				NC		NC	
Aromatic solvents								
No exposure[c]	860	(97.8%)	7651	(97.9%)	Ref		Ref	
Very low	9	(1.0%)	47	(0.6%)	1.70	0.83 – 3.49	2.25	1.03 – 4.90
Low	6	(0.7%)	62	(0.8%)	0.86	0.37 – 2.00	0.75	0.31 – 1.80
Medium	<3				NC		NC	
High	<3				NC		NC	
Chlorinated solvents								
No exposure[c]	836	(95.1%)	7513	(96.1%)	Ref		Ref	
Very low	25	(2.8%)	187	(2.4%)	1.20	0.79 – 1.84	1.42	0.91 – 2.23
Low	10	(1.1%)	60	(0.8%)	1.50	0.76 – 2.94	1.07	0.53 – 2.17
Medium	7	(0.8%)	54	(0.7%)	1.17	0.53 – 2.57	1.12	0.49 – 2.56
High	<3				NC		NC	
Stoddard solvents								
No exposure[c]	859	(97.8%)	7658	(98.0%)	Ref		Ref	
Very low	4	(0.5%)	47	(0.6%)	0.76	0.27 – 2.11	0.54	0.19 – 1.55
Low	13	(1.5%)	76	(1.0%)	1.53	0.84 – 2.76	1.27	0.68 – 2.38
Medium	<3				NC		NC	
High	<3				NC		NC	

Totals do not add up due to missing data. [a] = one month before conception through three months after conception, [b] = adjusted for maternal age at delivery as a continuous variable (no missing values), [c] = no exposure for outcome under analysis.

Table 5 Estimated frequency of maternal occupational solvents exposure during the periconceptional period[a] and risk of gastroschisis in offspring, National Birth Defects Prevention Study, USA, 1997-2011

	**Gastroschisis cases (n= 879)**		**Total Controls (n = 7817)**		**Unadjusted**		**Adjusted[b]**	
**Solvent classes**	N	(%)	N	(%)	OR	95% CI	OR	95% CI
Any solvent								
No exposure	815	(92.7%)	7238	(92.6%)	Ref		Ref	
0 -10 hours per week	52	(5.9%)	471	(6.0%)	0.98	0.73 – 1.32	1.07	0.78 – 1.45
>11 hours per week	23	(1.4%)	108	(1.4%)	0.99	0.54 – 1.80	0.77	0.41 – 1.44
Aromatic solvents								
No exposure[c]	860	(97.8%)	7651	(97.9%)	Ref		Ref	
0 -10 hours per week	16	(1.8%)	133	(1.7%)	1.07	0.63 – 1.81	1.23	0.71 – 2.15
>11 hours per week	3	(0.3%)	30	(0.4%)	0.89	0.27 – 2.92	0.85	0.24 – 2.95
Chlorinated solvents								
No exposure[c]	821	(93.6%)	7311	(93.6%)				
0 -10 hours per week	48	(5.5%)	428	(5.5%)	1.00	0.73 – 1.36	1.05	0.76 – 1.45
>11 hours per week	8	(0.9%)	73	(0.9%)	0.98	0.47 – 2.03	0.70	0.33 – 1.50
Stoddard solvents								
No exposure[c]	859	(97.8%)	7658	(98.0%)	Ref		Ref	
0 -10 hours per week	12	(1.4%)	115	(1.5%)	0.93	0.51 – 1.69	0.75	0.40 – 1.40
>11 hours per week	7	(0.8%)	43	(0.6%)	1.45	0.65 – 3.24	1.06	0.46 – 2.46

Totals do not add up due to missing data. [a] = one month before conception through three months after conception, [b] = adjusted for maternal age at delivery as a continuous variable (no missing values), [c] = no exposure for outcome under analysis.
